# Survival of Adult Generated Hippocampal Neurons Is Altered in Circadian Arrhythmic Mice

**DOI:** 10.1371/journal.pone.0099527

**Published:** 2014-06-18

**Authors:** Brooke D. Rakai, Michael J. Chrusch, Simon C. Spanswick, Richard H. Dyck, Michael C. Antle

**Affiliations:** 1 Department of Psychology, University of Calgary, Calgary, Alberta, Canada; 2 Hotchkiss Brain Institute, University of Calgary, Calgary, Alberta, Canada; 3 Department of Neuroscience, University of Calgary, Calgary, Alberta, Canada; 4 Department of Cell Biology and Anatomy, University of Calgary, Calgary, Alberta, Canada; 5 Pharmacology & Therapeutics, University of Calgary, Calgary, Alberta, Canada; McGill University, Canada

## Abstract

The subgranular zone of the hippocampal formation gives rise to new neurons that populate the dentate gyrus throughout life. Cells in the hippocampus exhibit rhythmic clock gene expression and the circadian clock is known to regulate the cycle of cell division in other areas of the body. These facts suggest that the circadian clock may regulate adult neurogenesis in the hippocampus as well. In the present study, neurogenesis in the hippocampal subgranular zone was examined in arrhythmic *Bmal1* knockout (-KO) mice and their rhythmic heterozygous and wildtype littermates. Proliferation and survival of newly generated subgranular zone cells were examined using bromodeoxyuridine labelling, while pyknosis (a measure of cell death) and hippocampal volume were examined in cresyl violet stained sections. There was no significant difference in cellular proliferation between any of the groups, yet survival of proliferating cells, 6 weeks after the bromodeoxyuridine injection, was significantly greater in the *BMAL1*-KO animals. The number of pyknotic cells was significantly decreased in *Bmal1*-KO animals, yet hippocampal volume remained the same across genotypes. These findings suggest that while a functional circadian clock is not necessary for normal proliferation of neuronal precursor cells, the normal pruning of newly generated neurons in the hippocampus may require a functional circadian clock.

## Introduction

Proliferation of neural stem cells persists into adulthood in the subventricular and subgranular zones (SGZ) of the mammalian brain [1], [2]. The SGZ gives rise to new granule cells in the dentate gyrus (DG) of the hippocampus. Rates of proliferation and survival of these new neurons can be modulated by a number of factors, including the richness of environment, exercise, learning, sleep, stress, and levels of glucocorticoids [3]–[9]. Many of these factors are under circadian control, suggesting that the circadian clock may influence proliferation and survival of neurons formed during adulthood [10]. For example, manipulation of the cortisol circadian rhythm both enhances clock gene expression and suppresses mitosis in the DG [4]. Furthermore, the circadian clock may be involved in regulating the cell cycle in the SGZ, as disruption of the molecular clock leads to aberrant proliferation [11].

The master circadian clock is located in the suprachiasmatic nucleus (SCN) [12]. Circadian rhythms arise from autoregulatory transcription/translation feedback loops within individual cells. Specifically, a dimer of the CLOCK and BMAL1 proteins binds to an E-box element in the promoter regions of the *Period* genes *Per1* and *Per2* and the *Cryptochrome* genes *Cry1* and *Cry2* to initiate transcription. The protein products of the *Per* and *Cry* genes dimerize and translocate to the nucleus, where they inhibit the activity of CLOCK and BMAL1, thereby turning off their own expression. Gradual degradation of PER and CRY proteins over the night eventually releases CLOCK and BMAL1 from inhibition, allowing transcription to resume the following day [13]. Transgenic animals lacking either both *Period* genes or both *Crypotochrome* genes are arrhythmic [14], [15]. Mice lacking *Bmal1* are the only single-gene knockout (-KO) model that reliably leads to arrhythmicity in overt locomotor rhythms as well as cessation of oscillations of clock gene expression in the SCN [16].

Expression of these clock genes is not limited to the master pacemaker in the SCN; oscillators can be found elsewhere in the brain and body. Of these extra-SCN brain regions that express the various clock genes, the granule cells of the DG and pyramidal cells of CA1 and CA3 in the hippocampus have the highest level of expression of *Per1, Per2, Clock* [17]–[19] and *Bmal1* [20], and expression of these genes in the hippocampus is rhythmic [21]. Furthermore, *Per2* is expressed in proliferating cells in the SGZ, and the *Per2^brdm1^* mutation leads to enhanced proliferation [22]. In other tissues, a functional circadian clock is necessary for normal mitotic cell division [23], [24].

Given the role of the circadian clock in regulating the cell cycle, and the presence of clock genes in the hippocampus, it is possible that a functional circadian clock is essential for normal patterns of proliferation and survival of neurons born during adulthood. Using arrhythmic *Bmal1*-KO mice and littermate controls, we examined cell proliferation in the SGZ, and survival and apoptotic cell death in granule cell layer (GCL) of the hippocampus.

## Materials and Methods

### Animals

Male animals were generated from an in-lab breeding program founded by heterozygous breeders from Jackson Laboratories, MA, USA (strain: B6.129-Arntl^tm1Bra^/J). Heterozygous breeders were paired to produce wildtype (WT, +/+, n = 11), heterozygous (Het, +/−, n = 10), and KO (−/−, n = 11) animals for proliferation and survival studies. A further 9 animals (WT n = 2, Het n = 3, KO n = 4) were produced for behavioral recordings. Mice were weaned at 21–22 days of age, and were housed with same sex littermates in a colony room with a 12∶12 light/dark cycle (lights on at 7 AM). Animals were provided food and water ad libitum and all procedures were performed in accordance with the Canadian Council on Animal Care guidelines, and were approved by the Life and Environmental Sciences Animal Care Committee at the University of Calgary (Protocol # AC12-0003). All attempts were made to minimize pain and discomfort.

### Genotyping

Animals were genotyped according to the protocol of Bunger et al., [16] using multiplex PCR with one forward primer (CCACCAAGCCCAGCAACTCA) and two reverse primers, one specific to the portion of the *Bmal1* gene that is deleted in the KO animals (ATTCGGCCCCCTATCTTCTG) and the other specific to the Neomycin gene that replaces the deleted portion of the *Bmal1* gene in the engineered gene (TCGCCTTCTATCGCCTTCTTGACG). The first reverse primer amplifies a 400 bp band corresponding to the wild-type allele, while the second reverse primer amplifies a 600 bp band. PCR was performed on genomic DNA from tail biopsies for 40 cycles of 95°C, 15 s: 60°C, 15 s: 72°C, 1 min in 1× PCR buffer (Sigma-Aldrich) containing 3.5 mM MgCl_2_.

### Behavior

To confirm that *Bmal1*-KO mice were arrhythmic, animals (WT n = 2, Het n = 3, KO n = 4) were housed in cages equipped with running wheels to record circadian patterns of wheel-running behavior as has been described previously [25]. Animals were housed in a light-dark cycle for about 4 weeks, before being transferred to constant darkness for a further four weeks. A Chi-square periodogram was used to confirm the presence or absence of a significant circadian rhythm over the last 16 days in constant darkness. The power of the chi-square statistic for the dominant period was used for analysis, and if no significant rhythm was detected, a power of zero was assigned.

### Proliferation

Animals were injected intraperitoneally (i.p.) with 50 mg/kg bromodeoxyuridine (BrdU) (Sigma-Aldrich) on postnatal day 60 at midday. 24 hours later they were deeply anesthetized with sodium pentobarbital (240 mg/kg) and perfused transcardially with phosphate buffered saline (PBS) followed by 4% paraformaldehyde. Brains were postfixed in 4% paraformaldehyde for 24 hours, followed by at least 24 hours in 20% sucrose in PBS. Four series of 35 µm sections were collected throughout the extent of the DG using a cryostat.

Immunohistochemistry procedures were based on Antle and colleagues [26]. All washes were at room temperature unless otherwise noted. A diaminobenzidine procedure was used to assess proliferation as follows: tissue was rinsed in Tris-buffered saline (TBS) then in 0.5% H_2_O_2_ in 0.1%TBS for 10 minutes, then in 50% formamide/2X saline-sodium citrate for 2 hours at 65°C, then rinsed for 15 minutes in 2X saline-sodium citrate, then a 30 minute incubation in 2N HCl at 37°C. Sections then had a 10 minute 0.1 M borate buffer (pH 8.5) rinse. TBS rinses preceded 1-hour incubation in TBS-Plus (3% Normal rabbit serum solution of TBS and Triton X-100). Sections were incubated for 48 hours in sheep anti-BrdU (1∶1000, in TBS-Plus, AB1893, Abcam, ON, Canada) at 4°C. Next, TBS rinses preceded 1-hour incubation in biotinylated rabbit anti-sheep antibody (1∶200, Vector Labs, ON, Canada). TBS rinses preceded 1-hour avidin-biotin complex incubation (ABC elite, Vector Labs). TBS rinses preceded a 3-minute reaction in 25 ml TBS containing 0.05% diaminobenzidine (DAB), 0.02% NiCl_2_, and 80 µl of 30% H_2_O_2_. Sections were dehydrated in an alcohol series, mounted on slides, and coverslipped using Permount. The number of nuclei stained for BrdU in the SGZ was counted on thirteen sections. As this represented only every 4^th^ section, this final count was multiplied by 4 to provide an estimate of the total number of BrdU-labeled cells.

### Survival

Mice were injected with BrdU (100 mg/kg i.p.) at midday. Six weeks post-injection they were perfused and brain tissue was collected as described above. Immunohistochemistry was similar to that described above except for the following differences for immunofluorescence. The tissue was incubated in 10% normal goat serum, followed by 24 hours in TBS-Plus containing rat anti-BrdU (1∶200, Abd Serotech, Raleigh, NC, USA), and mouse anti-NeuN (1∶2000, Millipore, Billerica, MA, USA). Secondary antibody (1∶500, Alexa Fluor 488-labelled goat-anti-mouse, biotin-labelled goat anti-rat, Jackson ImmunoResearch, PA, USA) incubations were for 24 hours, and the biotin was labeled using a 1-hour Streptavidin Alexa Fluor 594 (1∶500, Life Technologies Inc., Burlington, ON, Canada) incubation. TBS rinses preceded mounting tissue on gel-coated slides. After brief air drying, tissue was coverslipped with Krystalon. BrdU-labelled cells were counted in all sections and the total was multiplied by 4 to obtain an estimate of total numbers expected in the entire DG. Using a Nikon C1si Spectral Confocal microscope, BrdU/NeuN co-localization was assessed across the rostral-caudal extent of the DG. A cell was considered positive for BrdU/NeuN if a nuclear signal from both the 488 nm and 561 nm lasers co-localized in the x-, y-, and z-axes.

### Apoptosis Analysis and Hippocampal Volume

One series of sections from the animals used for the survival of BrdU labelled cells was stained with cresyl violet to examine differences in numbers of pyknotic/apoptotic cells in the DG. A pyknotic cell was defined as one in which the nucleus exhibited the following features: strong and homogeneous staining, condensed chromatin and fragmentation of the nucleus [27]. The same series of tissue was also utilized to determine GCL volume using the Cavalieri method [28], [29]. Briefly, images of brain sections containing the DG were taken using a Zeiss Axioskop 2 microscope attached to a Qimaging camera using a 5X (NA 0.25) objective. ImageJ (http://rsbweb.nih.gov/ij/) was employed to position a random, systematic sampling grid over each image. An area per point of 0.01 mm^2^ was deemed appropriate, and the total number of points in contact with the GCL was counted in each section. The number of contact points per section was multiplied by the area associated with each point (0.01 mm^2^), the section cut thickness (35 µm), and the section sampling fraction (4). The resulting numbers were then summed to provide an estimate of total GCL volume.

### Statistical Analysis

All cell counting was performed blind to genotype. All data were analyzed using one-way analysis of variance (SigmaStat, v 2.03; Systat Software, Inc, IL, USA) with Tukey post hoc tests.

## Results

### Behavior

**Figure 1:**
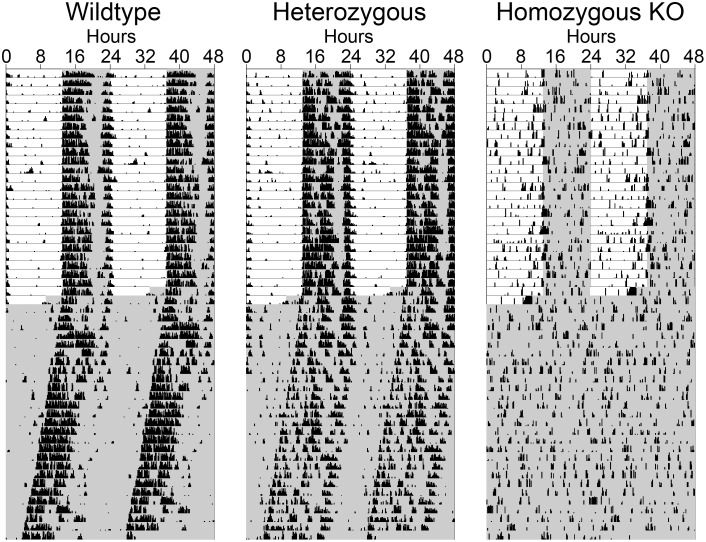
Homozygous *Bmal1*-KO mice are arrhythmic. Representative wheel-running activity charts (actograms) from *Bmal1* (+/+), (+/−) and (−/−) animals. Every horizontal line represents 2 days of activity, with subsequent days plotted to the right of and below the previous day. Vertical deflections from the horizontal represent 10 minute bins in which wheel revolutions were detected, with the height of the deflection being proportional to the number of revolutions. The animals were housed in a light/dark cycle for the first 4 weeks (darkness indicated by grey shading) and were then transferred into constant darkness for another 4 weeks. Wildtype (left) and heterozygous (middle) animals had normal circadian patterns of activity in both the light/dark cycle as well as in constant darkness. The *Bmal1*-KO mice were arrhythmic throughout. Animals in this circadian activity assessment were different than those used in the subsequent proliferation/survival experiments.

Using a Chi-square periodogram, *Bmal1*-KO mice had either no detectable rhythm (n = 2, Figure 1) or a rhythm outside the circadian range (19.0 h and 11.5 h, both with low power). *Bmal1*-KO mice had significantly lower power in their Chi-square periodograms (*F_(2,8)_* = 9.291, *p* = 0.015) than either wildtype or heterozygous animals.

### Proliferation

**Figure 2:**
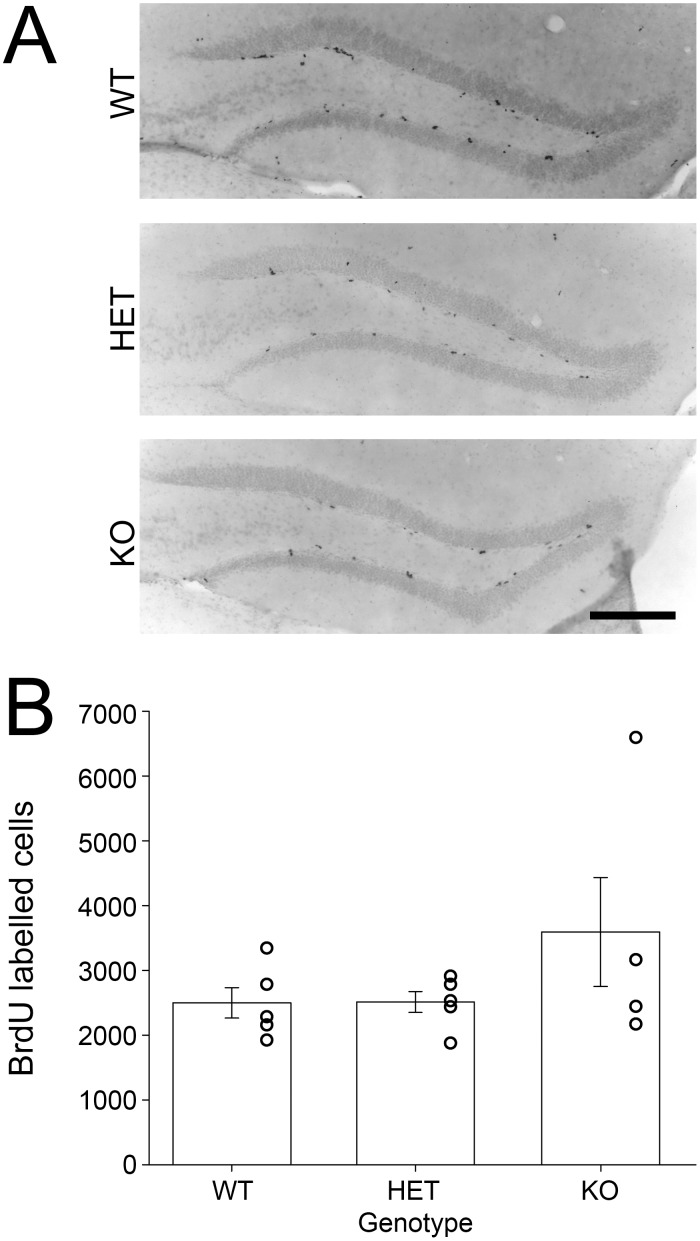
*Bmal1*-KO do not exhibit proliferation differences in the sub granular zone of the dentate gyrus. A) Photomicrographs of DAB labelled BrdU positive cells in *Bmal1* (+/+), (+/−) and (−/−) SGZ of the DG, 24 hours after an i.p. injection of BrdU. Scale bar = 200 µm. B) Number of cells labelled with BrdU throughout the extent of the hippocampus, calculated by counting cells in every fourth slice and multiplying by 4. Differences between genotypes were not significant.

There were no significant differences between genotypes in the number of BrdU-positive cells 24 hours after the BrdU injection (*F_(2,13)_* = 1.289, *p* = 0.314, Figure 2). A single *Bmal1*-KO animal had exceptionally high number of BrdU-labelled cells, leading to a high amount of variance, and a slight increase in the mean number of newly generated cells in the *Bmal1*-KO group. All other *Bmal1*-KO animals had virtually the same number of BrdU labeled cells as did wildtype and heterozygous animals.

### Survival

**Figure 3:**
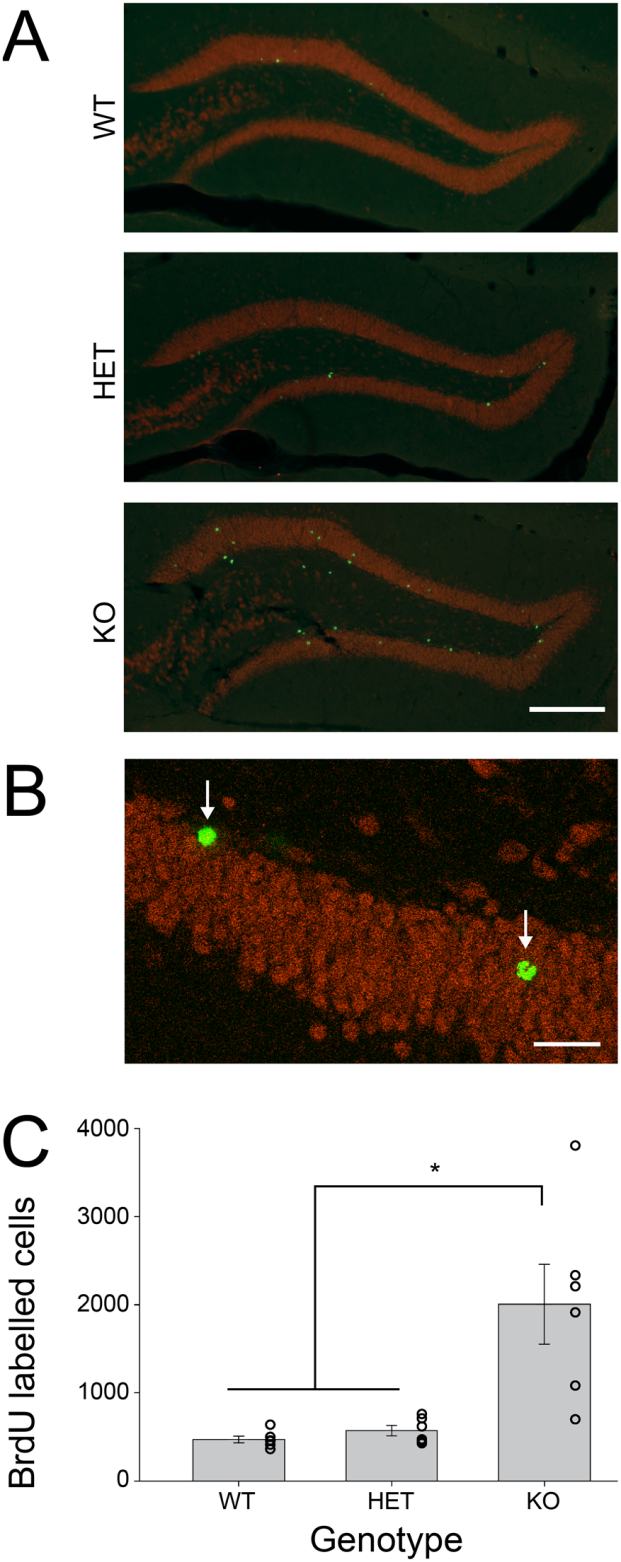
*Bmal1*-KO exhibit enhanced survival of newly generated cells in the dentate gyrus relative to heterozygous and wildtype mice. A) Representative photomicrographs of BrdU (green) and NeuN (red) immunoreactivity in *Bmal1* (+/+), (+/−), and (−/−) mice 6 weeks after an i.p. injection of BrdU. Scale bar = 200 µm. B) High-magnification confocal image of NeuN/BrdU labeled cells. Scale bar = 10 µm. C) Total number of BrdU labelled cells throughout the hippocampus, calculated by counting cells in every fourth slice and multiplying by 4. *p<0.05.

*Bmal1*-KO mice had significantly more BrdU labelled cells 6 weeks after BrdU injection (*F*_(2,15)_ = 10.882, *p* = 0.001, Figure 3). The number of BrdU positive cells did not differ between wildtype and heterozygous animals (*p* = 0.959). Confocal analysis revealed that the majority of BrdU-labeled cells were NeuN positive (WT = 94.5±2.1%; Het = 90.2±3.06%; KO = 93.07±2.1%) and there was no difference between the genotypes (*F*_(2,15)_ = 0.800, *p* = 0.46).

### Pyknosis and Hippocampal Volume

**Figure 4:**
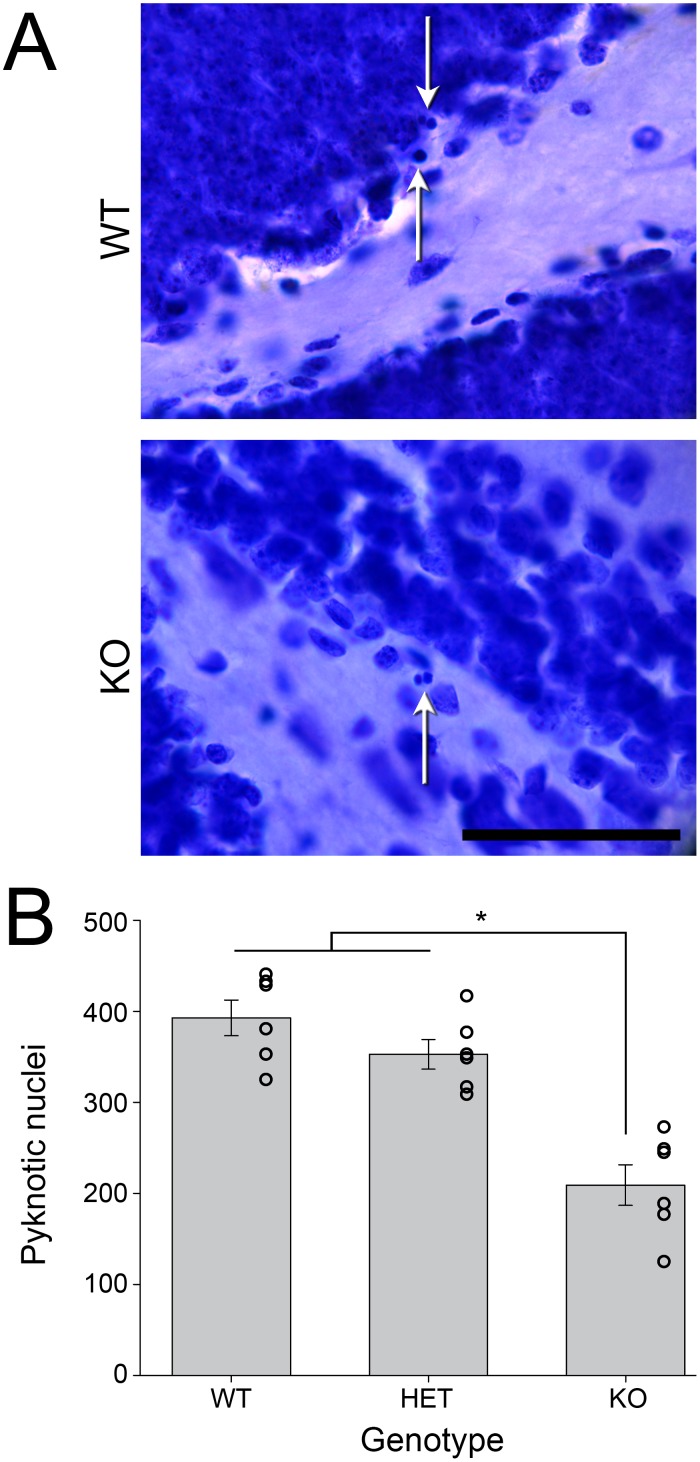
*Bmal1*-KO exhibit decreased pyknosis, indicative of diminished cell death, in the dentate gyrus relative to heterozygous and wildtype mice. A) Photomicrograph of cresyl violet stained pyknotic cells, shown by the white arrows. Scale bar = 50 µm. B) Estimates of the total number of GCL pyknotic cells throughout the extent of the hippocampi of *Bmal1* (+/+), (+/−), and (−/−) mice, calculated by counting pyknotic cells in every fourth slice and multiplying by 4. *p<0.05.

**Figure 5:**
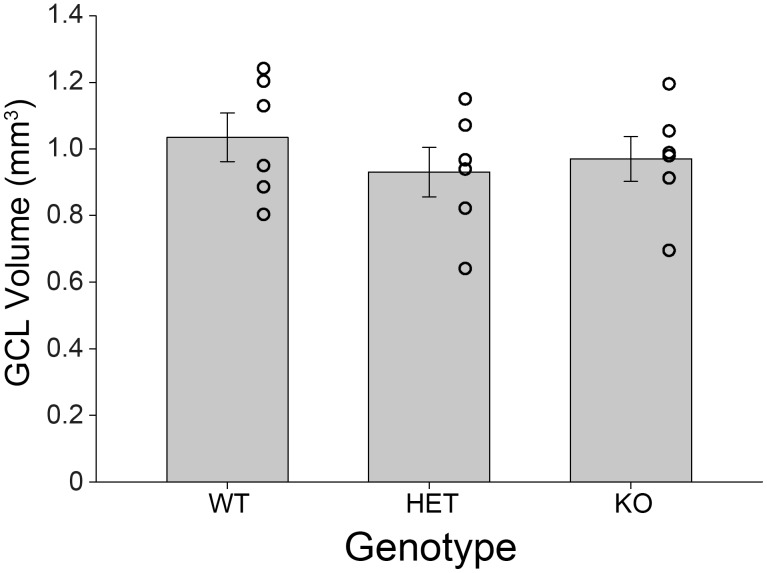
The volume of the hippocampus does not differ between wildtype, heterozygous and *Bmal1-*KO mice. A) Cavalieri estimates of total hippocampal volume in *Bmal1* (+/+), (+/−), and (−/−) mice at approximately 100 days of age. Estimates were calculated from the cresyl violet stained tissue series utilized in Figure 3 for pyknotic cell counts. Hippocampal volume estimates were not significantly different across genotypes.

*Bmal1*-KO animals had significantly fewer pyknotic cells in the DG than either the wildtype or heterozygous animals (*F*_(2,17)_ = 24.115, *p*<0.001, Figure 4). There was no difference between wildtype and heterozygous animals (*p* = 0.349). The GCL volume did not differ across genotypes (*F*_(2,17)_ = 0.534, *p* = 0.597, Figure 5).

## Discussion

The circadian clock has been suggested to regulate the cell cycle in both the brain [11], [22] and periphery [23], [24]. We show here that arrhythmic *Bmal1*-KO mice exhibit normal levels of proliferation in the SGZ, but enhanced survival relative to wildtype animals 6 weeks after labelling proliferating cells. Given that proliferation is no different, we confirmed a decrease in the number of pyknotic cells in the DG, indicative of decreased apoptosis, consistent with the observation of enhanced survival. Overall, these findings suggest that *Bmal1* is necessary for normal apoptotic pruning in the DG.

There are three possible mechanisms by which apoptosis could be impaired in *Bmal1*-KO mice. First, since circadian clock genes are rhythmically expressed throughout the hippocampal formation [17]–[22], [30]–[33], it is possible that it is the circadian rhythm generated within these cells that is essential for directing apoptosis. In an *in vitro* cancer model, disrupting the negative limb of the transcription translation feedback loop is associated with enhanced cell death [34], [35] Therefore, it might be expected that disruption *Bmal1* (i.e., the positive limb) could lead to lower cell death. Alternatively, cell survival/apoptosis may be affected by SCN-controled rhythms such as glucocorticoid levels or exercise [4], [9], [10]. However, exercise is unlikely to explain the enhanced survival observed, as *Bmal1*-KO mice exhibit lower, not higher, activity levels [16]. Finally, this may be a case of pleiotropy, where *Bmal1*’s role in regulating apoptosis may be separate and distinct from its role in regulating circadian rhythms. Many genes involved in growth and apoptosis are regulated by the transcription factor Myc, which binds to E-boxes to regulate gene expression [36]. If BMAL1 competes with Myc for binding to some of these E-box elements then removal of BMAL1 could lead to increased Myc binding to those E-boxes.

While the idea of pleiotropy is intriguing, a number of other studies have suggested that the circadian clock regulates the cell cycle and neurogenesis. There is a circadian rhythm in proliferation [37]. The period of the cell cycle in neural stem/progenitor cells (NSPCs) is approximately a day [38]. Exercise enhances neurogenesis, and the timing of voluntary exercise is controlled by the circadian clock [10]. Disruption of normal sleep wake patterns disrupts neurogenesis [39], although this is independent of disruption of the circadian clock [8]. One study has reported a daily rhythm in proliferation in the hippocampus, with higher proliferation during the day (rest phase), but only in the hilus where proliferation is predominantly gliogenic rather than neurogenic [40]. The hippocampus and the DG express a number of clock genes, including *mPer2* [18], [22]. This expression starts when cells are immature proliferating cells, and persists into adulthood. Proliferation is enhanced in mice in which the PAS domain of the *mPer2* gene has been deleted (*Per2^brdm1^*) [22] although after 3 weeks, the number of surviving neurons in these mice does not differ from wild type controls, owing to enhanced apoptosis [22]. The difference between the present study and those with the *Per2^brdm1^* mice may be due to a couple reasons. First, *Per2^brdm1^* mice are rhythmic, whereas *Bmal1*-KO mice are not. Alternately, PER2 is on the negative limb of the E-box mediated transcription-translation feedback loop, while BMAL1 constitutes the positive limb of these loops. If neurogenic or apoptotic factors were expressed in part due to activation/inactivation of E-boxes, one might expect the opposite results when disrupting the positive versus the negative limbs of the loop.

The role of *Bmal1* in cell proliferation is equivocal. A recent study reported enhanced proliferation in the SGZ in *Bmal1*-KO animals, and attributed this to enhanced entry and diminished exit from the cell cycle [11]. The opposite phenomenon is observed with *in vitro* cell culture models, where over-expression of *Bmal1* in has been associated with enhanced proliferation [41] and deletion of *Bmal1* leads to delayed proliferation of hepatocytes [23] To further complicate the picture, in the current study we found no effect on proliferation in the *Bmal1*-KO animals, the same genetic model used by Bouchard-Cannon et al. [11]. A number of differences between these studies could account for the disparate findings. First, Bouchard-Cannon et al. [11] labeled proliferating cells at the light-dark transitions, while we looked at midday. Although these animals are arrhythmic, the light-dark cycle may impose temporal organization on a variety of physiological processes. Furthermore, the ages examined may have contributed to the observed difference; our animals were 60 days of age when treated with BrdU, while those examined by Bouchard-Cannon et al. [11] were only 40 days of age. While proliferation decreases with age, prominent decreases are usually associated with much older animals than were examined in the present study [42], although *Bmal1*-KO mice do show some characteristics of accelerated aging [43], [44]. Finally, both Bouchard-Cannon et al. [11] and ourselves reported high variability in the proliferation of SGZ cells in *Bmal1*-KO mice. In our study, this was due to a single animal with high proliferation. Given the high variability and differences in phases, this issue needs to be re-examined more closely with more detailed sampling of clock phases.

While enhanced neurogenesis has generally been associated with improved performance on learning tasks [3], [45], [46], this may not be the case with *Bmal1*-KO mice. In fact, a recent report demonstrated diminished performance on a radial arm maze task, suggesting that the extra neurons impair performance [11]. As wildtype animals generally only retain a small percentage of newly generated neurons, it has been argued that only those cells that integrate into hippocampal circuits in a coherent fashion are retained, while the remaining are pruned through apoptosis [3], [45], [46]. If that is true, then the diminished cell death observed here might suggest that the newly generated cells cannot be pruned effectively and therefore they may be interfering with proper hippocampal functioning. Alternatively, it is possible that the deficit in learning arises independently of the altered survival of DG neurons. The circadian clock has been implicated in a variety of learning tasks, including both hippocampal-dependent and -independent tasks [47]–[49]. Thus arrhythmic *Bmal1*-KO mice may have impaired performance on these learning tasks due to their lack of rhythmicity, rather than altered survival of DG neurons. However, this possibility is less likely, as *Bmal1*-KO mice were only impaired in hippocampal-dependent tasks.
